# Protective Effects of *Borago officinalis* Extract on Amyloid ****β****-Peptide(25–35)-Induced Memory Impairment in Male Rats: A Behavioral Study

**DOI:** 10.1155/2014/798535

**Published:** 2014-06-11

**Authors:** Fatemeh Ghahremanitamadon, Siamak Shahidi, Somayeh Zargooshnia, Ali Nikkhah, Akram Ranjbar, Sara Soleimani Asl

**Affiliations:** ^1^Neurophysiology Research Center, Hamadan University of Medical Sciences, Hamadan 65178-3-8736, Iran; ^2^Student Research Committee, Hamadan University of Medical Sciences, Hamadan 65178-3-8736, Iran; ^3^Department of Toxicology and Pharmacology, School of Medicine, Hamadan University of Medical Sciences, Hamadan 65178-3-8736, Iran; ^4^Anatomy Department, School of Medicine, Hamadan University of Medical Sciences, Hamadan 65178-3-8736, Iran

## Abstract

Alzheimer's disease (AD) is a neurodegenerative disorder and most common form of dementia that leads to memory impairment. In the present study we have examined the protective effects of *Borago officinalis* (borage) extract on Amyloid **β** (A**β**)-Induced memory impairment. Wistar male rats received intrahippocampal (IHP) injection of the A**β**(25–35) and borage extract throughout gestation (100 mg/kg). Learning and memory functions in the rats were examined by the passive avoidance and the Morris water maze (MWM) tasks. Finally, the antioxidant capacity of hippocampus was measured using ferric ion reducing antioxidant power (FRAP) assay. The results showed that A**β**(25–35) impaired step-through latency and time in dark compartment in passive avoidance task. In the MWM, A**β**(25–35) significantly increased escape latency and traveled distance. Borage administration attenuated the A**β**-induced memory impairment in both the passive avoidance and the MWM tasks. A**β** induced a remarkable decrease in antioxidant power (FRAP value) of hippocampus and borage prevented the decrease of the hippocampal antioxidant status. This data suggests that borage could improve the learning impairment and oxidative damage in the hippocampal tissue following A**β** treatment and that borage consumption may lead to an improvement of AD-induced cognitive dysfunction.

## 1. Introduction

Alzheimer's disease (AD) is the most common cause of dementia that is estimated to affect approximately 36 million people worldwide [1]. AD is a disease that is commonly characterized by a gradual decline of memory, language, and cognitive ability. The nerve cells in the brains of Alzheimer's patients progressively shrink and die. Such neuronal cell death occurs first in the brain regions that are responsible for learning and memory, but it ultimately spreads to the entire brain [2]. It has been reported that the cholinergic system of Alzheimer's patients damages, resulting in decreased acetylcholine-producing choline acetyltransferase activity, decreased choline absorption, and decreased acetylcholine release, as well as decreased cortical acetylcholinesterase activity [3]. Cholinergic neurons of basal forebrain nuclei enter the hippocampus and cerebral cortex, and these neurons are crucial for memory, concentration, and other cognitive procedures [4].

Senile plaques and neurofibrillary tangles are the hallmark pathological features that are observed in the cortex, hippocampus, basal forebrain, and amygdala of an Alzheimer's patient [5]. Neurofibrillary tangles are intracellular fibrillar aggregation of the microtubule-associated protein tau which is hyperphosphorylated and oxidized. Senile plaques consist of insoluble fibrillar amyloid *β* (A*β*). A*β* destabilizes cellular Ca^2+^ homeostasis, consequently, inhibits hippocampal long-term potentiation, and disrupts synaptic plasticity [6, 7]. In addition, A*β* induced elevation of reactive oxygen species (ROS) levels in neurons resulting in protein oxidation, lipid peroxidation, ROS formation, and cellular dysfunction, leading to calcium ion accumulation and subsequent neuronal death [8–10]. Furthermore, A*β* causes damage to mitochondrial membranes and hence increases the amount of intracellular H_2_O_2_, thus affecting the genes downstream by interacting with numerous receptors and damaging neurons, ultimately accelerating cell death and hippocampus alteration [11–13].

The hippocampus is a relevant structure that is highly involved in cognition and psychological function and there is evidence that this structure is rapidly and extremely affected by an injection of the A*β* fragment (A*β*25–35) in rat [14]. As mentioned above, oxidative stress and inflammation following A*β* involve development and progression of AD. Antioxidants that prevent the detrimental consequences of A*β* are consequently considered to be a promoting approach to neuroprotection in AD brain [15].* Borago officinalis*, also known as borage, is a plant with nutritional value that is also used in traditional medicine in Iran. It has been known for its mood elevating properties as early as the first century A.D. [16].

Dietary use of borage exhibited immune-modulator [17] and blood pressure lowering effects in normal and hypertensive rats [18]. Borage oil has been promoted as an effective treatment for different pathologies, such as diabetic neuropathy and rheumatoid arthritis [19, 20].

Several experimental studies have shown that borage has antioxidant properties by decreasing the level of oxidative stress and free radical scavenging activity [21, 22].

Phytochemical studies reveal that borage contains tannins, resine, ascorbic acid, beta carotene, niacin, riboflavin, rosmarinic acid, and flavonoids [22, 23]. Borage is considered one of the best sources of gamma-linolenic acid (GLA) [24] which is known to have beneficial effects on brain ageing. It has been reported that treatment of aged rats with GLA restores the hippocampal LTP [25] and improves both memory and N-methyl-D-aspartic acid receptor function [26].

As the use of traditional medicine is widespread and plants still represent a large source of natural antioxidants, we seek to determine if borage extract as the best source of both GLA and antioxidant capacity can improve the cognitive and memory ability on A*β*-induced learning and memory deficits in rat.

## 2. Materials and Methods

The A*β*(25–35) was purchased from sigma-Aldrich company (St Louis, MO, USA).* Borago officinalis* leaves were obtained in dried condition from Research Institute for Islamic and Complementary Medicine (Tehran, Iran). A*β*25–35 was solubilized in sterile water at 1 *μ*g/*μ*L concentration and stored at −20°C.

### 2.1. Animals

We included 28 male Wistar rats (Pasteur-Iran), weighing 250–300 g in this experimental study. All animals were group-housed and given ad libitum access to food and water. Housing conditions were maintained at a temperature of 21 ± 2°C and the relative humidity of 50 ± 5% on a 12 h light/12 h dark cycle.

The rats randomly were assigned to the following groups:

 the control or intact group (*n* = 7) that was left undisrupted;
 the sham-operated group;
 the A*β*25–35 model group which received single bilateral intrahippocampal (IHP) injections of A*β*25–35 [27];
 the borage-treated group that received borage extract (orally, 100 mg/kg) following IHP injection of A*β*25–35 for 14 days [28].

### 2.2. Preparation of Borage Extract

Dried borage leaves were cleaned and ground into coarse powder by electrically driven device. The powdered material was soaked into aqueous ethanol (80%) for one week with occasional shaking [29]. The extract was filtered through a Whatman filter paper and evaporated to dryness under reduced pressure at a maximum of 40°C using a rotary evaporator.* Borago officinalis* yielded 10.9% dried extract. The extract was completely dissolved in distilled water and kept at 4°C.

### 2.3. Intrahippocampal Injection of A*β*25–35

The animals were anesthetized with the ketamine (100 mg/kg) and xylazine (10 mg/kg) and transferred to a stereotaxic apparatus (Stoelting, Wood Dale, IL, USA). Injection was made using a 10 *μ*L microsyringe (Hamilton-Reno, NV, USA). Relative to the bregma and with the stereotaxic arm at 0°, the coordinates for the dentate gyrus were posterior −3.6; lateral ±2.3; and dorsal 3 mm [30].

**Figure 1:**
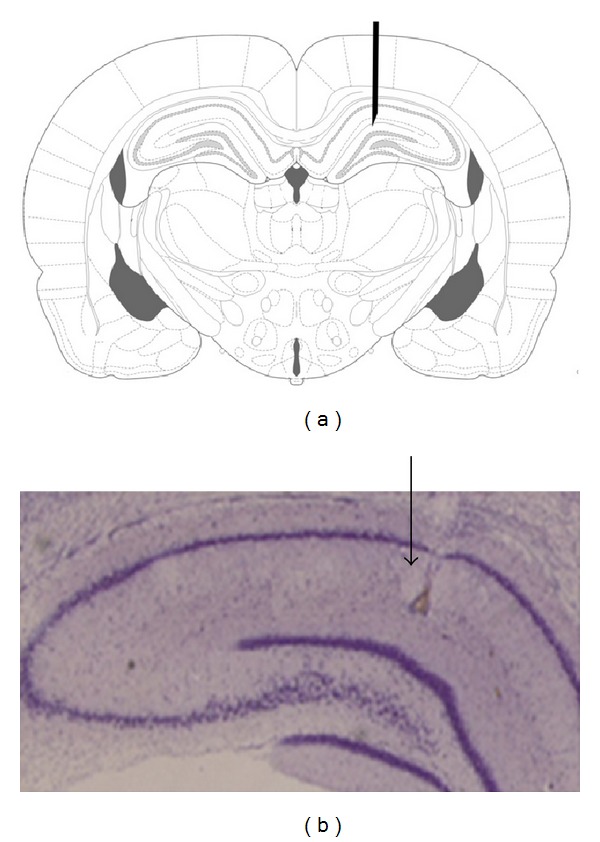
Schematic (a) and cresyl violet stained (b) photograph representing the microinjection site of Amyloid *β* into the hippocampus (black arrow).

A*β* solution (6 *μ*L) was bilaterally injected into the region over 1 *μ*L/2 min. the cannula was left in place for 2 min after each injection to allow for diffusion. Sham operated rats received vehicle solution. The skin was then sutured and the animals were left to recover in a warm box before returning to their home cages. The injection site was checked by injection trypan blue instead of peptide in preliminary experiments (Figure 1).

### 2.4. Passive Avoidance Learning

The passive avoidance test was started two weeks after the A*β* injection. The apparatus consisted of two chambers of the same size (20 × 20 × 30 cm). The chambers were separated by a guillotine door. The walls and floor of one compartment consisted of white opaque resin and the walls of the other one were dark. An intermittent electric shock (100 V, 0.3 mA, and 0.5 s) was delivered to the grid floor of the dark compartment by an isolate stimulator [14].

Each rat was gently placed in the white compartment and after 5 s the guillotine door was opened and the animal was allowed to enter the dark module. Immediately after entering the dark chamber, the door was closed and an electric shock was delivered through the floor grid (acquisition trial). Then the rats were returned to their home cages. Twenty-four hours later, each rat was again placed in light chamber (retention trial). The interval between the placement in the light chamber and the entry into the dark chamber (STL) and the total time spent in dark compartment (TDS) were recorded in the absence of electric foot shocks, as indicator of inhibitory avoidance behavior.

### 2.5. Assessment of Spatial Memory

As described previously [31], spatial memory testing was carried out using Morris water maze (MWM) apparatus. The MWM consisted of a circular pool (180 cm in diameter, 60 cm in depth) painted black and filled to a depth of 35 cm with water at a temperature of 22 ± 1°C. Numerous visual cues were present around the room and remained constant during the length of the experiment. The pool was divided into four quadrants with four starting locations, which were referred to as north (N), east (E), south (S), and west (W), each located at equal distances along the pool rim. An invisible platform (10 cm diameter) was located 1 cm below the water in the center of the northern quadrant that remained consistent for all animals across the training trials. The rats were trained between 10:00 a.m. and 12:00 noon for four days. Training consisted of two blocks with four trials. During training, each animal was allowed to swim until they located the hidden platform or until 90 s has elapsed. All groups were trained from each of the starting positions (N, E, S, and W). There was a 30 sec period between the two trials, which was spent on the platform. Rats were allowed to rest for 5 min between two consecutive blocks. Installed above the pool was a video camera (Nikon, Melville, NY, USA) linked to a tracking system to record a number of parameters including the time taken to reach the hidden platform (escape latency) and the length of the swim path (traveled distance). On day 5, a probe trail was performed in which the platform was removed from the pool and each rat was allowed to swim for 60 s. For these probe trails, percentage of time spent in the target quadrant was recorded.

### 2.6. Histological Verification

For verification of injection position using a light microscope (Olympus, Japan), the trypan blue injected rats were perfused with 4% paraformaldehyde in 0.1 M phosphate buffer (pH = 7.3) and the hippocampi were serially sectioned into 10 *μ*m coronal sections by a microtome (Leica Instruments, Germany). After deparaffinization and rehydration, sections were stained in 0.1% cresyl violet for 3 minutes. Finally, the sections were photographed with a digital camera (Olympus, DP 11, Japan) attached to a microscope (Olympus Provis, Ax70, Japan) and the stained slices were qualitatively analyzed for the injection site.

### 2.7. Ferric Reducing/Antioxidant Power (FRAP) Assay

After memory assessment, the animals were decapitated, brains were removed, and the extracted hippocampi were immediately frozen in liquid nitrogen and maintained at −80°C until processing.

The hippocampus portion was gently homogenized in ice-cold phosphate buffered saline (0.1 M, pH 7.4) to give a 10% homogeny suspension and used for FRAP assay.

Briefly, 50 *μ*L of homogenate was added to 1.5 mL freshly prepared and prewarmed (37°C) FRAP reagent (300 mM acetate buffer (pH = 3.6), 10 mM TPTZ in 40 mM HCl, and 20 mM FeCl_3_·6H_2_O in the ratio of 10 : 1 : 1) in a test tube and incubated at 37°C for 10 min. The absorbance of the blue colored complex was read against reagent blank (1.5 mL FRAP reagent + 50 *μ*L distilled water) at 593 nm. Standard solutions of FeII in the range of 100 to 1000 mM were prepared from ferrous sulphate (FeSO_4_·7H_2_O) in distilled water. FRAP values were expressed as nmol ferric ions reduced to ferrous form/mg tissue.

### 2.8. Statistical Analysis

Data were presented as mean ± S.E.M and analyzed by SPSS version 16 software. The data of the escape latency and traveled distance during the training days were analyzed using two-way analysis of variance (ANOVA), treatment as one factor and training days as the second factor. Statistical analyses of the FRAP value, passive avoidance test, and percent of time spent in target quarter of probe trial were performed using one-way ANOVA. Tukey multiple comparison tests were used to analyze the significance of the differences between the groups, when appropriate. Value of *P* < 0.05 was considered significant.

## 3. Results

Histological analysis showed that injection of A*β* was in their desired location, according to the atlas of Paxinos and Watson (Figure 1) [30].

### 3.1. Passive Avoidance Task

**Figure 2:**
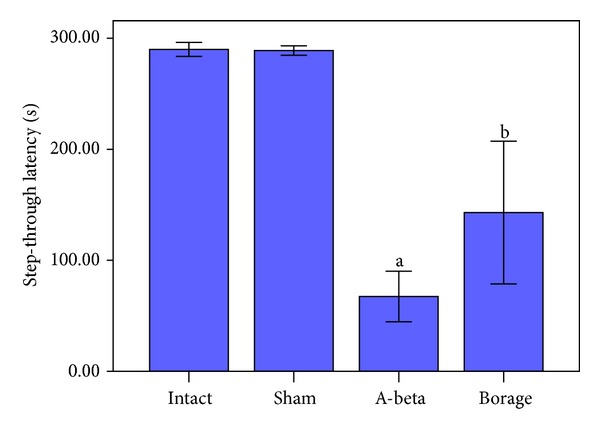
The mean of the step-through latency in the passive avoidance task. Vertical bars show S.E.M. (a *P* < 0.001 versus intact and sham groups; b *P* < 0.01 versus A-beta group).

In the acquisition trial (14 days after the operation), we found no difference between the intact and other groups in the step-through latency (STL, data not shown). However, the IHP injection of A*β*(25–35) reduced STL in the retention trial compared to intact and sham groups (*P* < 0.01, Figure 2).

**Figure 3:**
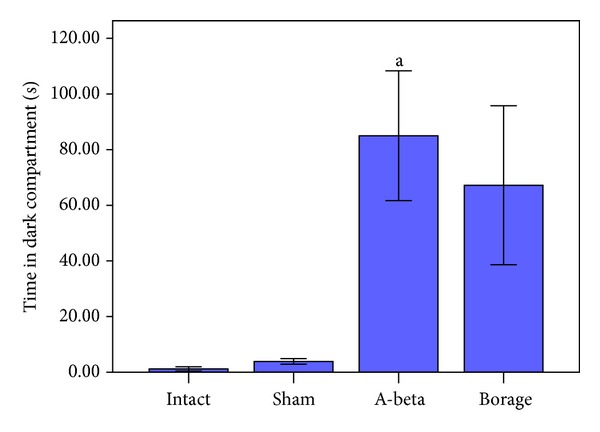
The mean of the time spent in dark compartment in the passive avoidance task. Vertical bars show S.E.M. (a *P* < 0.001 versus intact and sham groups).

Furthermore A*β*-treated rats spent more time in dark compartment (TDC) in respect to intact and sham-operated rats (*P* < 0.001, Figure 3). Administration of borage for 14 days caused a significant increase in STL compared with A*β*-treated rats (*P* < 0.01, Figure 2). Borage treatment insignificantly attenuated the TDC when compared to A*β* group (Figure 3).

### 3.2. MWM Performance

**Figure 4:**
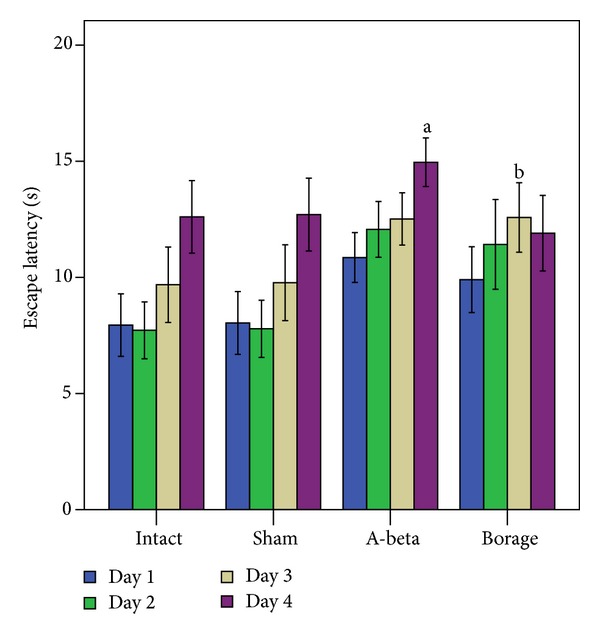
The mean of latencies to find hidden platform in the MWM. Each block represents the average latency of four consecutive trial days. Data present as mean ± S.E.M. (a *P* < 0.001 versus intact and sham groups; b *P* < 0.01 versus A-beta group).

A two-way analysis of variance revealed significant effects of treatment [F (3, 28) = 26.85, *P* < 0.001] and training days [F (3, 24) = 7.8, *P* < 0.001]. In addition, there was a significant interaction between treatment and training days [F (9, 72) = 2.35, *P* < 0.05]. Analysis of the four training days showed that intact group spent less time to find the hidden platform (escape latency) than the other groups (Figure 4) and this time was more in the first day compared to other days. Longer escape latency indicates more sever spatial memory deficits. The post hoc analysis indicated a significant difference between the intact and sham-operated groups and the rats which received A*β* (*P* < 0.001). According to the results, borage administration caused significant reduction in escape latency compared with the A*β*-treated group (*P* < 0.01).

**Figure 5:**
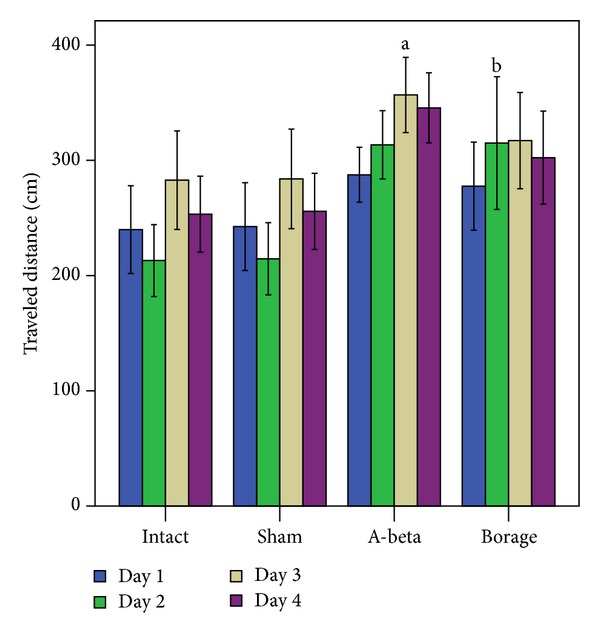
The mean of traveled distance in the MWM. Each block represents the average of traveled distance of four consecutive trial days. Data present as mean ± S.E.M. (a *P* < 0.001 versus intact and sham groups, b *P* < 0.001 versus A-beta group).

In accordance with the latency data, there was a significant effect of treatment [F (3, 28) = 11.23, *P* < 0.001] and training days [F (3, 55) = 21.78, *P* < 0.001] on the traveled distance. There was no significant interaction between training days and treatment. As shown in Figure 5, a significant difference in traveled distance was seen between A*β*-treated rats and intact group (*P* < 0.001). A*β*-treated rats that received* Borago* for 14 days showed less traveled distance compared with A*β* group (*P* < 0.001).

**Figure 6:**
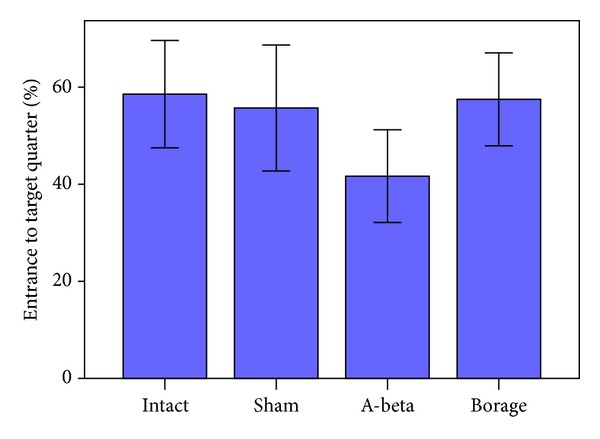
The mean of the percent of entrance to target quarter in the probe trial in the MWM. Data present as mean ± S.E.M.

Percentage of the entrance to target quadrant in the probe trial session was investigated. Results showed that the intact group spent more time in target quadrant (5.87 ± 553) than other groups (sham: 5.57 ± 649; A*β*: 4.67 ± 333;* Borago*: 5.57 ± 0.479, Figure 6).

### 3.3. FRAP Assay

**Figure 7:**
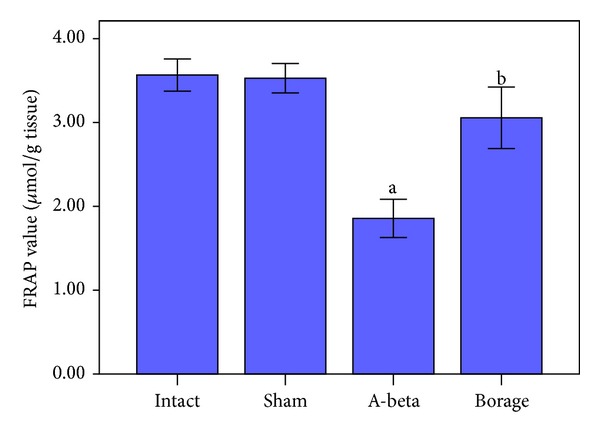
Effect of borage on antioxidant power (FRAP value) of hippocampus homogenate samples following microinjection of A*β* into rat hippocampus. Data present as mean ± S.E.M. (a *P* < 0.001 versus intact and sham groups; b *P* < 0.05 versus A-beta group).

A*β* caused a significant reduction in FRAP value of homogenate samples as compared with intact and sham-operated animals (*P* < 0.001, Figure 7). Borage treatment increased antioxidant power (FRAP value) of brain homogenate samples (*P* < 0.05, Figure 7).

## 4. Discussion

The major finding of this study was the attenuation of learning and memory impairment by borage following IHP injection of A*β*. Treatment with borage was protective against A*β*-induced oxidative stress in the hippocampus. A*β* plays an important role in the pathophysiology of Alzheimer disease and a close correlation exits between A*β* procedure and the neurodegeneration process of AD [32]. Nitta et al. showed that the performance of the water maze task in *β*-amyloid-treated rats was impaired and choline acetyltransferase activity significantly decreased in the frontal cortex and hippocampus [33]. The deposition of *β*-amyloid protein in the brain is related to the impairment of learning and cholinergic neuronal degeneration and the *β*-amyloid protein-treated rats could be used as animal model for AD [14]. The key brain regions involved in navigation in the MWM task include the striatum, the frontal, and spatially the hippocampus [34, 35]. In the present study, the bilateral IHP injection of A*β*(25–35) induced a significant learning disturbance in the passive avoidance and MWM tasks in the rat. Consistent with our results several experimental studies have shown that the infusion of A*β*(25–35) into the brain induced learning impairment in the passive avoidance and radial-arm maze tasks [14] and memory disturbance in the Y-maze, passive avoidance, and water maze tasks [36]. Similarly, Harkany et al. reported that bilateral injection of A*β*(25–35) in rat induced learning deficits in passive avoidance tasks [37]. Our results of FRAP assay showed that A*β*(25–35) could decrease antioxidant power of the hippocampus. The reaction of FRAP assay is linearly related to molar concentration of the antioxidant(s) present. Consistent with our study several lines of evidence suggest that the A*β* is inserted into the neuronal membrane bilayer and generates oxygen-dependent free radicals and then causes the lipid peroxidation and protein oxidation [38, 39]. Furthermore, the A*β* deposition activates the acute immune response of microglial cells and astrocytes and leads to production and activation of inflammation-related proteins such as complement factors, cytokines, such as interleukin 1, interleukin 6, and tumor necrosis factor *α*, thus leading to synaptic damage, neuronal loss, and the activation of other inflammatory participants [40–42]. The brain is sensitive to oxidative stress because of low antioxidant and cell membrane lipid [43]. Therefore, the use of different spices and aromatic herbs as external antioxidant is one of the most common therapeutic strategies for treatment of neurological disease [44, 45] and improvement of brain damage and cognitive function [31, 46, 47]. The present study demonstrated that administration of borage was protective against A*β*-induced memory and antioxidant deficit. As expected following A*β* injection, a significant reduction in antioxidant power, as indicated by FRAP value, was observed. Borage increased the antioxidant power of homogenate samples of hippocampus which was consistent with results from the study by others that extracted antioxidants from borage [48, 49]. Our results showed that borage oil could improve A*β*-induced memory impairment. The protective effect of borage on memory can be related to its function of scavenging free radicals and to its high content of GLA [24].

Indeed, borage's hydroalcoholic extract inhibits linolenic acid oxidation, liposome peroxidation, and/or scavenges 2,2-diphenylpicrylhydrazyl (DPPH) radical in vitro [21]. Several studies evaluated the relationships between antioxidant activity of borage extract and its GLA content [50, 51].

Tasset-Cuevas et al. have shown that both borage seed oil and GLA exert a role in the genomic stability, acting as desmutagenic agents against hydrogen peroxide by scavenging the ROS originated by the model genotoxicant used [52].

Similarly, Duffy et al. observed an increase in learning ability after administration of evening primrose oil which is rich in GLA following uteroethanol exposure in rats [53].

Treatment with GLA rich natural oils was previously shown to partially prevent nerve ischemia and associated conduction anomalies and improved long-term potentiation in the hippocampus in rats with experimental diabetes mellitus [26, 54].

Kavanagh et al. have shown that GLA treatment increased anti-inflammatory cytokines in hippocampus of lipopolysaccharide-treated rats and suggested that these effects may be coupled with fatty acid-induced upregulation of peroxisome proliferator-activated receptor gamma which processes known anti-inflammatory effects [55].

Taken together, it is possible that borage oil inhibits ROS generation, scavenges free radicals, inhibits the effects of inflammatory proteins, suppresses the inhibitory effects of A*β* on learning, and might be implicated in the protection of A*β*-induced neurotoxicity.

It is not clear whether the borage extract or its active ingredient crosses the blood brain barrier and that needs to be investigated further.

## 5. Conclusion

In summary, we demonstrated that the IHP injection of A*β*(25–35) induced a significant reduction in antioxidant power and learning deficits in passive avoidance and Morris water maze tasks and borage administration significantly attenuated traveled distance and escape latency reduction following injection of A*β*(25–35) and improved A*β*-induced time spent deficiencies. Borage treatment improved step-through latency and time spent in dark compartment in passive avoidance tasks. Also, borage increased the antioxidant power of homogenate samples of hippocampus. Therefore, it is likely that borage may be useful to treat patients with impaired memory function.
